# Progressive and disseminated histoplasma infection and hemophagocytic lymphohistiocytosis in an immunocompetent adult

**DOI:** 10.1002/ccr3.2079

**Published:** 2019-03-27

**Authors:** Khalid M. Dousa, Alejandro De la Hoz, Elizabeth Church, Tiffany Onger, Federico Perez, Elie Saade

**Affiliations:** ^1^ Division of Infectious Diseases and HIV Medicine University Hospitals Cleveland Medical Center Cleveland Ohio; ^2^ Division of Infectious Diseases and HIV Medicine, Department of Medicine Case Western Reserve University School of Medicine Cleveland Ohio; ^3^ Grupo de Investigación en Enfermedades Infecciosas, Hospital Universitario San Ignacio Pontificia Universidad Javeriana Bogotá Colombia; ^4^ Department of Medicine University Hospitals Cleveland Medical Center Cleveland Ohio; ^5^ Division of Infectious Diseases and HIV Medicine, Louis Stokes Cleveland VA Medical Center Case Western Reserve University School of Medicine Cleveland Ohio

**Keywords:** hemophagocytic lymphohistiocytosis, *Histoplasma* spp, histoplasmosis, systemic lupus erythematosus

## Abstract

Hemophagocytic lymphohistiocytosis (HLH) in adults is a life‐threatening underdiagnosed disorder that complicates different infectious syndromes and overlaps with sepsis. No guidelines are available for the management of HLH in adults. A high index of suspicion is required in patients with disseminated histoplasmosis.

## 1 INTRODUCTION

Secondary hemophagocytic lymphohistiocytosis (HLH) is a life‐threatening immune‐mediated disease often triggered by infection, hematologic neoplasias, or autoimmune disorders. It is characterized by inappropriate activation of cytotoxic T cell and natural killer cells, leading to macrophage activation in the bone marrow and phagocytosis of other blood cells.^1^ Clinically, this results in fever, hepatosplenomegaly, and cytopenia. Associated infections include HIV, EBV, CMV, other herpes viruses, influenza, parvovirus B19, viral hepatitis, *Mycobacterium tuberculosis*, *Rickettsia* species, staphylococcal species, *Escherichia coli*, leishmania, malaria, toxoplasmosis, and *Histoplasma *spp infection. The overall incidence is low, estimated at approximately 1 per 800 000 person‐years.^2^ Diagnostic criteria include fever, splenomegaly, cytopenia of at least 2 cell lines, hypertriglyceridemia and/or hypofibrinogenemia, hemophagocytosis on pathology, low natural killer cell activity, ferritin>500 ng/mL, or increased soluble CD25 concentration >2400 U/mL. Patients must meet 5/8 criteria to be diagnosed

Hemophagocytic lymphohistiocytosis is a diagnostic challenge as the syndrome is often mistaken for sepsis. Delay in treatment of the underlying can be fatal. Histoplasma infection leading to HLH is rare, and there is no consensus on the treatment strategy. There are case reports and case series in the literature describing ~27 cases of histoplasmosis leading to secondary HLH. The majority of cases are reported in immunosuppressed patients, either with HIV or with biologic therapy for underlying autoimmune disease. Case reports have described treatment with amphotericin alone, as well as with adjunct steroids, IVIG, or etoposide.

## 2 CASE

**Figure 1:**
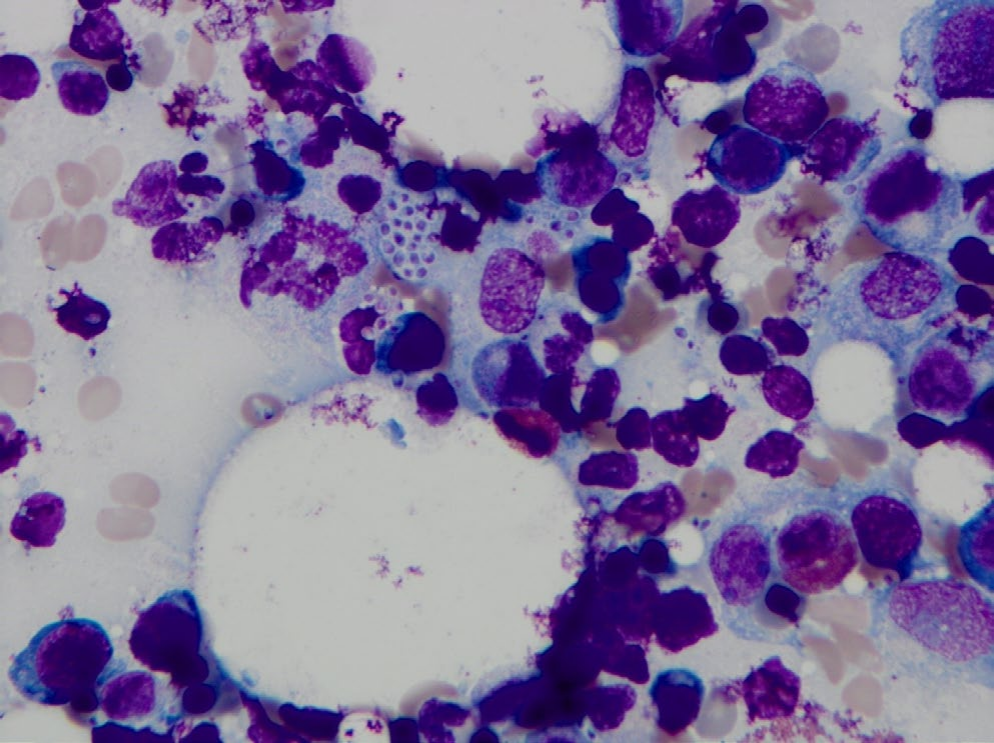
Wright‐Giemsa stain of the bone marrow biopsy specimen showing activated macrophages, including some with hemophagocytosis

**Figure 2:**
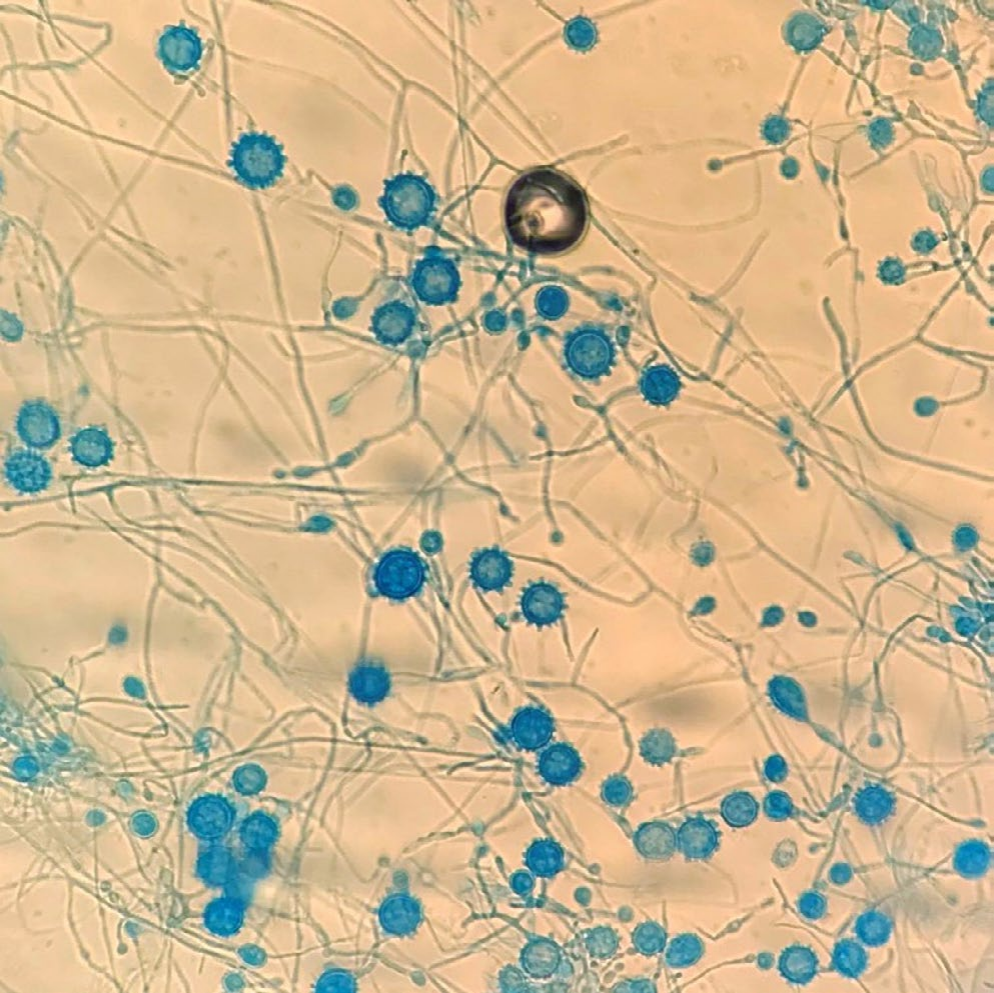
Bone marrow biopsy showing numerous yeast forms and spiked spherical conidia of *Histoplasma capsulatum* (lactophenol cotton blue stain)

A 60‐year‐old female presented with 3‐month history of intermittent fever, cough, dyspnea, fatigability, and personality changes. Prior to onset of symptoms, the patient reported exposure to decayed wood in the summer as her husband was remodeling their house. Her history was notable for systemic lupus erythematosus (SLE), diagnosed 15 years earlier. Although the patient denied follow‐up for SLE, she stated she was well controlled with hydroxychloroquine. A complete blood count showed cytopenia with hemoglobin 8.9 g/dL, white blood cells count of 1900 cells/mm^3^, and platelets count of 22 000 cells/mcl. Additional laboratory studies showed lactate dehydrogenase (535 unit per liter; normal range [140‐280]), serum triglycerides (259 milligrams per deciliter; normal range [<150]), ferritin (21 900 nanograms per milliliter; normal range [20‐500]), and fibrinogen (57 mg per deciliter; normal range [150‐400 mg per deciliter]), elevated hepatic transaminases, positive histoplasma antigen in the cerebrospinal fluid analysis and a negative HIV test. Computed tomography showed bilateral lung ground glass opacity with enlarged mediastinal lymph node and massive splenomegaly. Histologic examination of the bone marrow showed activated macrophages, including some with hemophagocytosis (Figure 1), intracellular numerous yeast form, and spiked spherical conidia of *Histoplasma capsulatum* (Figure 2). The patient received a diagnosis of HLH and disseminated histoplasmosis. Treatment with IV liposomal amphotericin B 5 mg/kg once daily was initiated for 4 weeks followed by oral itraconazole 100 mg a day for one week and then 200 mg daily to complete one year of therapy.^13^ Although she received a 10‐day course of dexamethasone 10 mg twice a day with improvement of the fever, they were stopped for agitation status. Her fever resolved 2 weeks after treatment along with all other symptoms. At follow‐up 5 months later, no new symptoms were reported, and the laboratory studies were normal.

## 3 DISCUSSION

Hemophagocytic lymphohistiocytosis in adults is considered one of the most critical clinical disorders, where high index of suspicion and early therapy are needed. In severe HLH, the presentation often overlaps with overwhelming sepsis, and the diagnosis of the true syndrome is often delayed resulting in multiorgan failure. Up to date, there is no definitive diagnostic test and the diagnosis is based on clinical findings or in autopsy. One key diagnostic clue is the extreme elevation in ferritin. While ferritin is often elevated in sepsis as well, levels >3000 should prompt consideration for other syndromes, including HLH.^3^ Early treatment of the infection trigger is recommended. The most common infection to trigger HLH in United States is EBV. Currently, there are no guidelines for treatment of HLH in adult population and most of the available recommendations for therapy is populated from pediatric population, which recommend administration of etoposide, dexamethasone, and liposomal amphotericin B (LAmB) for treatment of both conditions. In bacterial infections, particularly MTB, there is some evidence that treatment of the underlying cause is sufficient for resolution with or without HLH‐directed therapy in 60%‐78% of cases. Most of secondary HLH cases published in the literature are associated with HIV and particularly with low CD4 count, autoimmune disease on monoclonal antibody biologic drugs or immunosuppressive therapy, solid organ transplant recipients, and hematological malignancies. One case series and review of literature discussed 27 cases of histoplasmosis‐associated HLH, nine cases were seropositive for HIV, and most the remaining patients were all on immunosuppressive therapy, the only two patients with no underlying risk factor died despite therapy with amphotericin and corticosteroids.^4^

Herein, we report an adult presented with disseminated and progressive nature of *Histoplasma *spp infection who had not received any form of immunosuppressive medication and recount the complete resolution of the clinical syndrome and biomarkers without therapy directed toward HLH. The patient continued to do well with no clinical or biochemical relapse. If guidelines for treatment of HLH‐associated histoplasmosis in pediatric population were applied on our patient, etoposide would have been administered. This case highlights the need for high index of suspicion to diagnose HLH and call for necessity to develop guidelines for management of HLH‐associated infections in adult population. Special attention to disseminated *Histoplasma *spp infection as a possible trigger for HLH, irrespective of immune status, should be considered in patients living or travelled to an area endemic of histoplasmosis.

## CONFLICT OF INTEREST

The authors declared no conflict of interests.

## AUTHOR CONTRIBUTION

KD: structured the discussion and took care of the patient. ADH and FP: structured the discussion. EC, TO, and ES: structured the case description and took care of the patient. All authors reviewed and approved the final version of the manuscript.
